# Address of Welcome to the Dentists in Attendance upon the Seventeenth Annual Meeting of the Southern Dental Association

**Published:** 1885-07

**Authors:** L. A. Thurber

**Affiliations:** New Orleans


### ADDRESS OF WELCOME TO THE DENTISTS IN ATTENDANCE UPON THE SEVENTEENTH ANNUAL MEETING OF THE SOUTHERN DENTAL ASSOCIATION.

BY L. A. THURBER, D. D. S., NEW ORLEANS.

*Mr. President and Gentlemen:*

By request of the committee of arrangements, I have the distinguished honor of welcoming you to New Orleans; to the same hall where, fifteen years ago was held a meeting of the Southern Dental Association of the most gratifying character. Year after year we have seen this body steadily working to advance the destinies of a profession which honors every one of its members. To crown its efforts in the path of progress, this session is held in this Crescent City, chosen at this time by the civilized world as the grand exchange and point of display of all that can improve and benefit the condition of man. As dentists, as men proud of our calling, we have a right to a place in this congress of human ingenuity. It is appropriate that we should meet here and show the world the accomplishments of an art founded on the highest principles governing science, and made famous by American brains and American hands Ignored by the learned professions less than forty years ago, we, to-day, stand on a footing of equality with any craft whose mission is the alleviation of human suffering. And more than this, we are a recognized, independent, liberal profession. Our meetings are always fruitful of rich results. The more advanced tender freely the knowledge they possess, the experience they have gained, to the more humble or less privileged, with a generosity and kindness characteristic of members of one family. To a gathering of such loyal men I am here to say WELCOME! Welcome with the hope that the deliberations held here will add one more stone to that monument which you are erecting to your profession, and which is consigned to immortality.

To you who have assigned me this honorable task, I will only say, if I have not welcomed your visitors in language more eloquent, with thoughts of greater depth, I indulge the hope that you will forgive my short-comings, and only remember the sincerity of my purpose.

